# Two-Dimensional EspEn: A New Approach to Analyze Image Texture by Irregularity

**DOI:** 10.3390/e23101261

**Published:** 2021-09-28

**Authors:** Ricardo Espinosa, Raquel Bailón, Pablo Laguna

**Affiliations:** 1 Department of Biomedical Engineering, Universidad ECCI, Bogotá 111311, Colombia; 2 Biomedical Signal Interpretation & Computational Simulation (BSICoS) Group, Aragón Institute of Engineering Research (I3A), IIS Aragón, University of Zaragoza, 50018 Zaragoza, Spain; rbailon@unizar.es (R.B.); laguna@unizar.es (P.L.); 3 CIBER en Bioingeniería, Biomateriales y Nanomedicina (CIBER-BBN), 50018 Zaragoza, Spain

**Keywords:** image processing, texture, entropy, two-dimensional data, EspEn, irregularity

## Abstract

Image processing has played a relevant role in various industries, where the main challenge is to extract specific features from images. Specifically, texture characterizes the phenomenon of the occurrence of a pattern along the spatial distribution, taking into account the intensities of the pixels for which it has been applied in classification and segmentation tasks. Therefore, several feature extraction methods have been proposed in recent decades, but few of them rely on entropy, which is a measure of uncertainty. Moreover, entropy algorithms have been little explored in bidimensional data. Nevertheless, there is a growing interest in developing algorithms to solve current limits, since Shannon Entropy does not consider spatial information, and SampEn2D generates unreliable values in small sizes. We introduce a proposed algorithm, EspEn (Espinosa Entropy), to measure the irregularity present in two-dimensional data, where the calculation requires setting the parameters as follows: *m* (length of square window), *r* (tolerance threshold), and ρ (percentage of similarity). Three experiments were performed; the first two were on simulated images contaminated with different noise levels. The last experiment was with grayscale images from the Normalized Brodatz Texture database (NBT). First, we compared the performance of EspEn against the entropy of Shannon and SampEn2D. Second, we evaluated the dependence of EspEn on variations of the values of the parameters *m*, *r*, and ρ. Third, we evaluated the EspEn algorithm on NBT images. The results revealed that EspEn could discriminate images with different size and degrees of noise. Finally, EspEn provides an alternative algorithm to quantify the irregularity in 2D data; the recommended parameters for better performance are *m* = 3, *r* = 20, and ρ = 0.7.

## 1. Introduction

Image-processing applications have allowed the advance of several technologies in medicine, informatics, microscopy, agriculture, and others. Over time, various issues in science and technology have prompted improvements in algorithms for extracting features from digital images, which are useful in face detection, character recognition, and augmented reality [1]. Therefore, processing techniques let us handle the digitized images mathematically to obtain quantitative data and perform detection, recognition, segmentation, and classification tasks in order to obtain high-quality products while reducing time and costs in production [2].

Currently, texture is a crucial feature that represents an active interest in computer vision systems. Although there is currently no consensus on the formal definition of texture, it has been related to the surface of an object or phenomenon of repetitive pattern in images [3]. In fact, texture elements (called texels) give us information on the spatial distribution of local intensity variations of pixels in a neighborhood [4].

Moreover, texture analysis has been applied in artificial vision [5,6,7,8] and additive technology [9], since it provides content information (foreground objects, background, or noise), so it has helped the industrial sector getting better results in either identification or shape manufacture. As a result, texture analysis has had an important role in tracking video objects, vegetation, medical imaging, and remote sensing [10].

In the different fields of medicine, texture studies integrate the micro- and macro-structures of healthy and unhealthy tissues, where computer systems are used to reduce the human error factor [11]. For instance, variations on image texture have been quantified through regularity measurements, such as in dermatology, to categorize skin lesions [12,13]; magnetic resonance images to recognize and differentiate brain tumors from other tissues, such as gray matter, white matter, and cerebrospinal fluid [14]; identification lesions in parotid salivary glands [15]; and stability in dental implants [16].

Nevertheless, objective texture assessment is a current challenge. Although different alternatives have been proposed, there is not enough popular and widely applicable texture descriptor for images with different sizes, considering grayscale with and without noise. Haralick proposed a set of descriptors to evaluate the statistical texture properties of images, such as correlation, variance, entropy, uniformity, and homogeneity [17,18,19]. Correlation is a measure of the linear dependence of the gray level between pixels at specific positions. The variance (level of contrast) corresponds to the homogeneity and local variations. Entropy (irregularity) determines uncertainty (statistical randomness), which is used to depict the image texture. The uniformity (energy) describes the coincidence of the second angular momentum and corresponds to the sum of the square of the gray level co-occurrence matrix elements [20].

Entropy in images has had different applications, such as segmentation, filtering, and obtaining specific information. The entropy filter replaces the intensity of pixels by entropy values, in whose process Shannon’s Entropy algorithm is applied, where it is required a structured element with a certain size to be evaluated locally. Tsallis entropy [21] and Kapur entropy [22] have been set to segment foreground and background objects that require a threshold as the optimal separation value by maximizing the entropy function.

Although the entropy analysis on images is usually done by using Shannon theory, other bidimensional algorithms have been recently adapted to evaluate textures through irregularity measures which can be promising and helpful at the research approach. As highlighted in Silva et al. (2014), Two-Dimensional Sample Entropy analysis (SampEn2D) is an effective method to extract features from histological images of aging rat sural nerve to classify groups through measures of irregularity. They concluded that the elderly rat image is more regular than that of a young rat. As a result, SampEn2D can be a complementary method to morphometric indices [23]. Subsequently, da Silva et al., in 2016, tested their method in simulated images, texture database, and biological images of rat sural nerves again. They got a robust irregularity measure, considering SampEn2D properties and parameters: bidimensional matrix (*u*), length patterns that are square windows (*m*), and threshold (*r*) [24]. However, when SampEn2D is tried in small sizes, it can generate less reliable or undefined values. Moreover, it is too slow for most real-time applications. Hence, challenges to overcome using this entropy are still in force.

There is a growing interest in developing algorithms to measure irregularity in two-dimensional data, some based on existing ones, to solve current problems or limits, since they could provide relevant information for classification or detection tasks of useful patterns in fields such as agriculture, industry, and biomedical.

In this study, we propose an algorithm called Espinosa Entropy (EspEn) to measure the irregularity present in an image or two-dimensional data in general. We compared EspEn’s performance against other popular entropy estimators in image processing (Shannon Entropy and SampEn2D). Finally, we study EspEn’s sensitivity when we vary certain values of parameters that must be previously considered. Although there are other recent algorithms of two-dimensional entropy, such as distribution entropy (DistrEn2D) and dispersion entropy (DispEn2D), and despite their interesting results, we are not going to compare EspEn with DistrEn2D, because it is more focused on small-sized textures. Furthermore, mixing random values with an image does not significantly change the value of DistrEn2D.

This document has the following structure. In Section 2, two of the most popular entropy algorithms in image processing (Shannon Entropy and SampEn2D) and the EspEn algorithm proposal are presented. Section 3 describes the methodology used to evaluate the performance of the proposed algorithm and compares it to the other algorithms’ performance. Section 4 presents the results and discussions. Finally, Section 5 contains the conclusion of the study.

## 2. Shannon Entropy, SampEn2D, and EspEn Concepts

### 2.1. Shannon Entropy

Shannon Entropy is considered a measure of uncertainty related to the probability distribution that has been used as a Haralick descriptor to categorize the texture of the image. The normalized histogram is an intensity function that shows the count of pixels with equal intensity regardless of position. Entropy is the amount of individual information weighted by the probability of elements occurrence [25] and is defined as follows:

(1) $$Entropy=\underset{i}{\sum }p_{i}\;log_{2}p_{i}$$

The probability of occurrence for each intensity of gray is *p_i_* = *g/N*, where *g* represents each value of the histogram, and *N* represents the positions in the matrix.

### 2.2. SampEn2D Entropy

SampEn2D is an extension of the SampEn algorithm in 1D, applied to images, that seeks to preserve the original proposal as a measure of irregularity [24]. SampEn2D algorithm considers an image *u*(*i*,*j*) with width, *W*, and height, *H*. Let *x_m_*(*i*,*j*) be the set of pixels that form a square of length *m*, with column range *j* to *j + m* − 1 and row range *i* to *i + m −* 1.

Let *Nm* be the number of square windows (*x_m_*(*i*,*j*)) within *u* that can be generated for both *m* and *m* + 1. This can be calculated by *Nm* = (*W − m*) *×* (*H − m*). Considering a threshold of similarity, *r*, SampEn2D is defined as follows:

(2) $$SampEn_{2D}\left(u,m,r\right)=-Ln\frac{U^{m+1}\left(r\right)}{U^{m}\left(r\right)},$$

where

(3) $$U^{m}\left(r\right)=\frac{1}{N_{m}}\overset{i=H-m,\;j=W-m}{\underset{i=1;j=1}{\sum }}U_{i,j}^{m},$$

(4) $$U_{i,j}^{m}\left(r\right)=\frac{\left[\#\;of\;x_{m}\left(a,b\right)\;|\;d\left[x_{m}\left(i,j\right),x_{m}\left(a,b\right)\right]\leq r\right]}{N_{m}-1}$$

and

(5) $$U^{m+1}\left(r\right)=\frac{1}{N_{m}}\overset{i=H-m,\;j=W-m;}{\underset{i=1;j=1}{\sum }}U_{i,j}^{m+1},$$

(6) $$U_{i,j}^{m+1}\left(r\right)=\frac{\left[\#\;of\;x_{m+1}\left(a,b\right)\;|\;d\left[x_{m+1}\left(i,j\right),x_{m+1}\left(a,b\right)\right]\leq r\right]}{N_{m}-1}$$

where a ranges from 1 to *H − m*, *b* ranges from 1 to *W − m*, (*a*, *b*) ≠ (*i*, *j*) to exclude self-matches, and *r* can be defined as a fraction of image standard deviation. The distance function, *d*, is defined by the following:

(7) $$d\left[x_{m}\left(i,j\right),x_{m}\left(a,b\right)\right]=\max\left(\left|u\left(i+k,j+l\right)-u\left(a+k,b+l\right)\right|\right)$$

where *k* and *l* range from 0 to *m −* 1.

### 2.3. Espinosa Entropy Proposal (EspEn) for 2D

On the one hand, although Shannon Entropy is often used as a measure of image irregularity [26,27,28,29], it does not take spatial information into account. Therefore, the entropy value in noisy images or grayscale images could be similar due to the histogram, even when their texture information is different. Therefore, estimates of entropy as irregularity in an image may be wrong. Furthermore, it does not consider the comparison between pixels; thereby, the user cannot set the comparison bearing in mind the characteristics and conditions of the image, which is actually an advantage of current entropy algorithms.

On the other hand, due to the popularity of SampEn in the analysis of temporary signals, the SampEn2D extension has had some visibility for the analysis of irregularity in images (details in References [24,30,31,32,33]); some new methods have incorporated into their algorithm the calculation of SampEn2D, generating interesting alternatives, such as multiscale entropy (MSE2D) and its variant ModMSE2D [34]. However, expanding the SampEn1D method into the world of 2D data analysis or imaging should mean additional considerations, such as the number of *m* points taken as a pattern for comparison in SampEn1D; typically, *m* = 2 or *m* = 3 is less than the number of points (pixels) taken as a pattern in the case of SampEn2D. Some researchers have analyzed *m* = 1, *m* = 2, and *m* = 3, representing a square window of *m*∗*m*; for instance, the case of *m* = 3 in SampEn2D indicates that 9 pixels are taken [24]. In the case of comparing a pattern of 9 pixels with another set of pixels of the same quantity within the image, 1 pixel may be different, while the remaining are the same; for this example, we would say that there is a similarity of 89% than the user could accept as a similarity. SampEn2D is too strict in the comparison, since, if at least 1 pixel is different in the comparison between the pattern and the rest of the pixels of the same dimensions, there is no similarity, being even more critical with *m* + 1. The above leads to the vector $U^{m}i,j\;\left(r\right)$ of zero, and therefore the final estimate of the SampEn2D is Infinite (Inf) or no data (NaN), as shown in Silva et al. [24] when the researchers evaluated SampEn2D for *m* = 3 in noisy images. They also refer to the role of *r* (tolerance threshold) and the different effects produced by the variation of *r* for 1D signals and 2D data, effects that could be more associated with problems in estimating the entropy of an image. In addition, we consider that, since *r* is linked to the standard deviation (std), in the case of image processing, especially for very low *r* values or low values of the standard deviation (image with a single gray level) could be close to zero, due to the nature of pixel values, integer values from 0 to 255. Consequently, a tolerance threshold of zero or simply a very small value would indicate that the comparison distance is very limited, by a few values of gray levels; an extreme case would be a tolerance threshold lower than 1, in whose case the vector $U^{m}i,j\;\left(r\right)$ would not exist.

At this point, a simplified entropy estimator is proposed, called EspEn (Espinosa Entropy), considering the relevant aspects of Shannon and SampEn2D: the comparison of patterns with the remaining of the pixels grouped in the same dimensions of the pattern proposed in SampEn2D, and the simplicity in calculating the probability of occurrence for each intensity of gray according to Shannon’s entropy. We tried to overcome the weaknesses of each algorithm to quantify the irregularity of an image.

#### EspEn Algorithm for Two Dimensions

**Figure 1:**
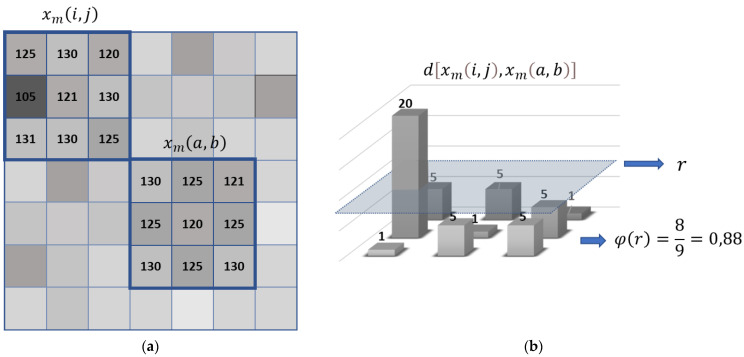
(**a**) Square windows example and (**b**) distances between two square windows.

EspEn is an estimator of the irregularity of an image that considers the probability of occurrence of a set of samples, with dimension $m^{2}$, that are similar within a similarity threshold *r*, with an acceptable percentage in the number of similar samples. The EspEn algorithm, similar to SampEn2D, considers an image *u*(*i*,*j*) with width, *W*, and height, *H*. Let *x_m_*(*i*,*j*) be the set of pixels that form a square window, with column range *j* to *j + m −* 1 and row range *i* to *i + m −* 1. The window construction would be *x_m_*(*i*,*j*) = [*u*(*i*,*j*), *u*(*i*, *j* + 1), …, *u*(*i*,*j + m −* 1), *u*(*i* + 1,*j*), *u*(*i* + 1,*j* + 1), …, *u*(*i* + 1,*j + m −* 1), …, *u*(*i + m −* 1,*j + m −* 1)]. Then, EspEn is defined by the following:

(8) $$EspEn\left(u,m,r\right)=-\ln(D^{m})$$

where

(9) $$D^{m}=\frac{1}{\left(H-m+1\right)\left(W-m+1\right)}\overset{i=H-m+1;j=W-m+1}{\underset{i=1;j=1}{\sum }}C_{i,j}^{m}$$

(10) $$C_{i,j}^{m}=\frac{\left[\#\;of\;\varphi \left(r\right)\geq \rho \right]}{\left(H-m+1\right)\left(W-m+1\right)-1}$$

where ρ is fixed and represents the percentage of similarity acceptable for the study, expressed in decimals.

(11) $$\varphi \left(r\right)=\frac{[\#\;of\;x_{m}\left(a,b\right)|d[x_{m}\left(i,j\right),x_{m}\left(a,b\right)]\leq r]}{m^{2}}$$

where 1 *≤ a ≤ H − m* + 1, 1 *≤ b ≤ W − m* + 1 y (*a*,*b*) ≠ (*i*,*j*) to exclude self-matches. The distance function, *d*, for EspEn is defined by the following:

(12) $$d\left[x_{m}\left(i,j\right),x_{m}\left(a,b\right)\right]=\left|u\left(i+k,j+l\right)-u\left(a+k,b+l\right)\right|$$

where *k*, *y*, and *l* vary from 0 to *m* − 1. Note that, in Equation (12), the maximum value of the distances is not estimated, but each of the distances calculated between the pattern and the set of pixels of the same dimensions is evaluated. In Figure 1a, an example of square windows is shown; *x_m_*(*i*,*j*) and *x_m_*(*a*,*b*) with *m* = 3 have different gray values. In Figure 1b, we see the distances between *x_m_*(*i*,*j*) and *x_m_*(*a*,*b*). Moreover, φ(r) is calculated by counting the distances within the threshold of similarity *r*; in the example, there are 8 distances ≤ *r*. This result is divided by the total number of possibilities ($m^{2}$); 0.88 is compared to ρ to establish the acceptable similarity between the windows, given by the observer.

The similarity threshold parameter (*r*) in EspEn should be fixed, considering the standard deviation of the image but not linked to it. We consider this parameter as an integer value that represents the variation of gray admitted in the study. More details of this parameter (*r*) are established in the Results and Discussions (Section 4.3.2).

## 3. Materials and Methods

### 3.1. Set of Images

This section describes the characteristics of the images used to analyze the performance of EspEn in evaluating the irregularity of an image. Synthetic images that had repeating (predictable) and clearly identifiable patterns (shapes) were created. These images were progressively contaminated with uniform white noise, similar to the process shown with *MIX*_2*D*_ in Reference [24], defined as follows:

(13) $$MIX{\left(p\right)}_{ij}=\left(1-p\right)X_{ij}+p\;Y_{ij}$$

where *X_ij_* represents the synthetic image, *Y_ij_* is the noise image with normalized random values with amplitude from 0 to 255 at each pixel with uniform distribution, and *p* represents the degree of contamination: *p* = 0 (without contamination) and *p* = 1 (only noise).

**Figure 2:**
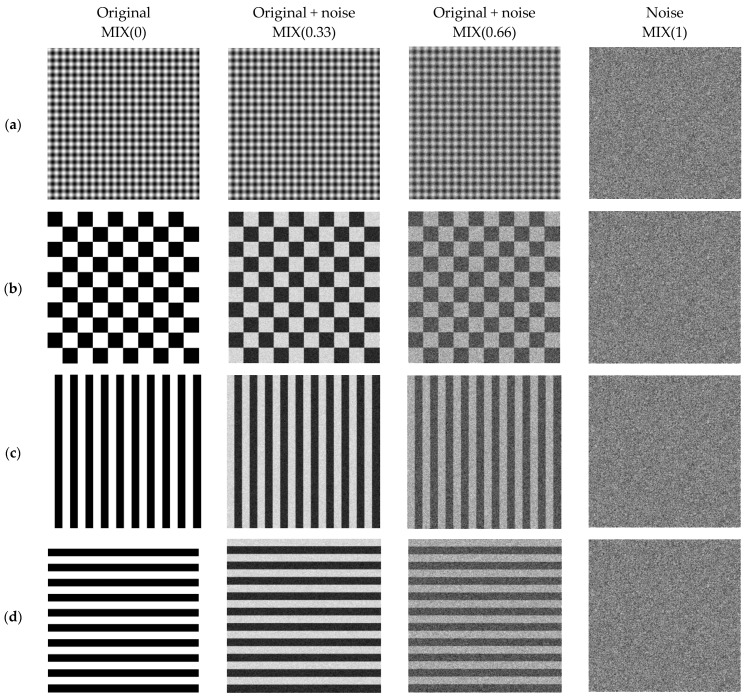
Synthetic images contaminated with different noise levels: (**a**) images based on sinusoidal functions, (**b**) chessboard, (**c**) vertical stripes, and (**d**) horizontal stripes.

Initially, four *X_ij_* images were generated with class unit8 and dimensions of 500 × 500 pixels, Figure 2a is based on sinusoidal functions created with the same process described in Reference [24], where *X_ij_* = *sin*(2*πi*/48) *+ sin*(2*πj*/48). Figure 2b is a checkerboard image with 50 black squares (pixels of value 0) and 50 white squares (pixels of value 255) interspersed; the box in the upper left corner is black, and the size of each box on the board is 50 × 50 pixels. Figure 2c represents vertical stripes, which were created by an automatic path in the matrix, each 50 columns, taking all the rows and making a displacement to replace the first 25 columns by pixels with value 255 (white) and pixels with a value of 0 (black) in the remaining 25 columns. This process was repeated until the full dimensions of the image were reached, thus obtaining 10 white stripes and 10 black stripes interspersed (each strip had 500 rows × 25 columns), starting with a white stripe. Figure 2d represents horizontal stripes, which were created through a cycle that ran through the matrix every 50 rows, selecting all the columns and performing an automatic scrolling to replace the first 25 rows by pixels with value 255 (white), and in the remaining 25 rows the pixels with a value of 0 (black). This process was repeated until the full dimensions of the image were reached, thus obtaining 10 white stripes and 10 black stripes arranged interspersed (each strip had 25 rows × 500 columns) and starting from a white strip. In each case, the complement images were considered to expand the set of images. There was a total set of 8 synthetic images.

Subsequently, a sampling (*s*) of the images was carried out with *s* = 1, 2, 3, 4, 5, and 10 in order to obtain images of 500 × 500, 250 × 250, 167 × 167, 125 × 125, 100 × 100, and 50 × 50 pixels, respectively. At every size change, 4 degrees of random noise contamination were considered *p* = 0, 0.33, 0.66, and 1. In this way, a total of 192 images were processed from (8 initial images) × (6 changes of *s*) × (4 changes of *p*) used to compare the results obtained by applying the entropy algorithms Shannon, SampEn2D, and the proposal EspEn.

### 3.2. Experiment and Parameters

Three experiments were performed: The first consisted of implementing 3 entropy algorithms (Shannon, SampEn2D, and EspEn) on synthetic images (MIX(0), MIX(0.33), MIX(0.66), and MIX(1)) with different sizes. The only input argument to the Shannon algorithm is the image. The parameters used in the SampEn2D algorithm were the image (*u*), the length of the square window (*m* = 2), and the tolerance factor or similarity threshold (*r* = 0.2 × standard deviation of each image). The parameters used in the EspEn algorithm were the image (*u*), length of the square window (*m* = 3), percentage of similarity between windows (*ρ* = 0.7), and the similarity threshold (*r* = 20).

The second numerical experiment was to implement the EspEn algorithm in synthetic images MIX(0), MIX(0.33), MIX(0.66), and MIX (1) with a size of 100 × 100 pixels and the value of the parameters *m*, *r*, and ρ to observe their influence or impact on the response of EspEn.

The third experiment consisted of implementing the EspEn algorithm with *m* = 3, *ρ* = 0.7, and *r* = 20, to 112 images from Brodatz’s database of normalized textures [35], sampled s = 6 to obtain images of size 107 × 107 pixels.

#### 3.2.1. Computational Cost: Shannon Entropy, SampEn2D, and EspEn vs. Image Size

Irregularity was measured in a set of images, using three entropy algorithms (Shannon, SampEn2D, and EspEn). The images used were synthetic images (described in Section 3.1) with dimensions of 500 × 500 pixels, which were sampled at *s* = 2, 3, 4, 5 and 10, obtaining image dimensions of 250 × 250, 167 × 167, 125 × 125, 100 × 100, and 50 × 50 pixels, respectively. The time elapsed during the execution of the algorithms in this procedure was measured.

The algorithms and numerical experiments described in the Methodology section were implemented by using MATLAB version R2018b, on a laptop with Intel Core i5-8250U 1.60 GHz CPU, 8 Gb RAM, and a Windows 10 operating system.

#### 3.2.2. EspEn and Dependence on *m*, *r*, and *ρ*

The images used in this numeral had dimensions of 100 × 100 pixels of MIX(0), MIX(0.33), MIX(0.66), and MIX(1). Total images used: 32, corresponding to 8 MIX images (4 initials and 4 with inverted color) × 4 degrees of contamination for each image (see details of the images in Section 3.1).

Comparisons of EspEn entropy values between groups of MIX images were performed with the Kruskal–Wallis test, due to the small sample size. Significance was set at *p* < 0.05.

##### EspEn and Dependence on the Length of the Square Window (*m*)

The algorithm parameters *ρ* = 0.7, *r* = 20 were fixed, and the value of *m* was changed (*m* = 1, 2, 3, 4, and 5) to evaluate the impact of *m* on the calculation of EspEn.

##### EspEn and Dependence on the Threshold of Similarity (*r*)

The algorithm parameters *m* = 3, *ρ* = 0.7 were fixed, and the value of *r* was changed (*r* = 5, 15, 25, 35, 45, and 55) to evaluate the impact of *r* on the calculation of EspEn.

##### EspEn and Dependence on the Percentage of Acceptable Similarity (*ρ*)

The percentage of similarity between windows was modified, namely *ρ* = 0.5, 0.6, 0.7, 0.8, 0.9, and 1, while the other parameters of the algorithm were fixed: *m* = 3 and *r* = 20.

#### 3.2.3. EspEn (*m*, *r*, and *ρ*) Applied to Images from Normalized Brodatz’s Textures Database

The normalized Brodatz texture database (NBT) contains images with different shapes and textures, where the spectral informational background of the grayscale Brodatz textures was removed so that the discrimination of the texture does not depend on the background information, using first-order statistics [35]. The set of images has been used in investigations related to the analysis of texture and irregularity; some investigations in which this database has been used are References [34,36,37].

We applied the EspEn algorithm with parameters *m* = 3, *ρ* = 0.7, and *r* = 20 to images from the NBT database; we sampled *s* = 6 to obtain images with dimensions of 107 × 107 pixels, and 112 images were processed.

## 4. Results and Discussion

### 4.1. Computational Cost

**Figure 3:**
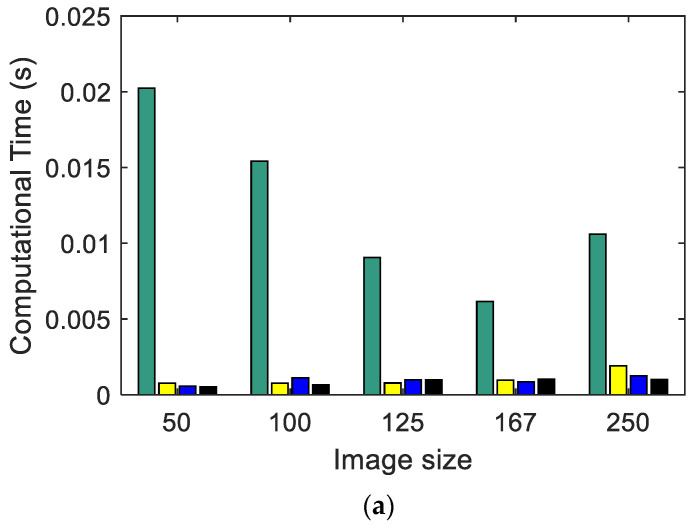

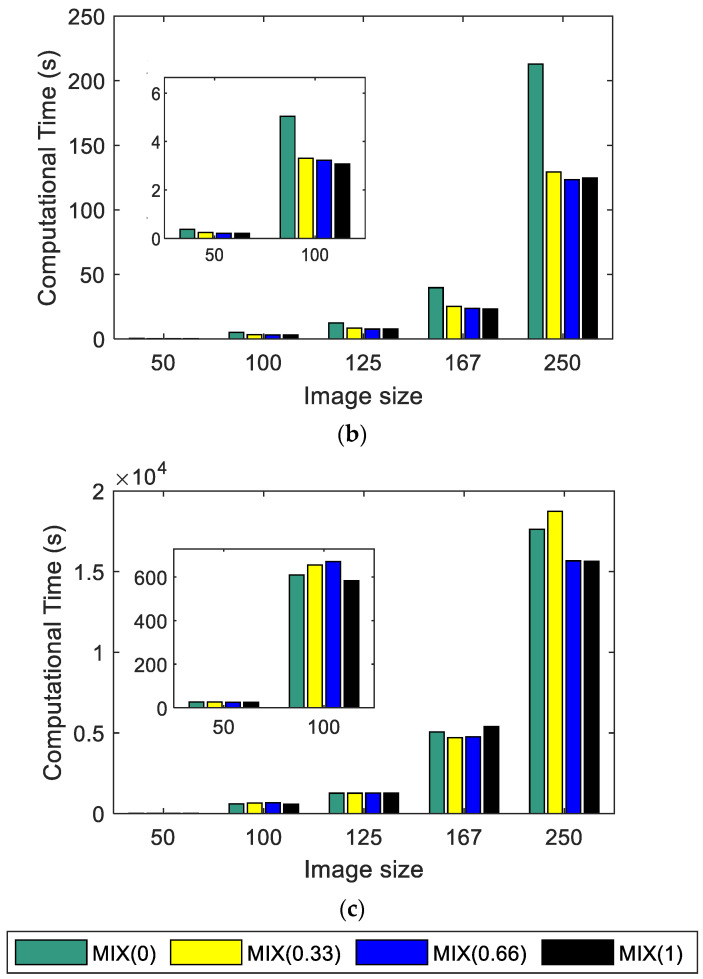
Computational time in seconds: (**a**) Shannon Entropy, (**b**) SampEn2D, and (**c**) EspEn.

Figure 3 shows the calculation time used by each entropy algorithm (Figure 3a Shannon, Figure 3b SampEn2D, and Figure 3c EspEn) applied to images with different sizes. The algorithm that used the least time was Shannon, and the algorithm that took the most time was EspEn, about 87 times more than SampEn2D. The images of 500 × 500 pixels could not be evaluated because the time used by EspEn exceeded 2 days of processing. Shannon and SampEn2D Entropy algorithms spent more time processing regular images (MIX(0)). SampEn2D and EspEn took more computation time as the images increased in size; this time used apparently increased exponentially as a function of size. Similar results were reported by da Silva et al. in 2018, when they used the multiscale entropy algorithm (MSE) adapted for two-dimensional data processing, which used SampEn2D as the basis for calculating irregularity [34].

The EspEn algorithm takes longer because it compares each possible pattern (*H − m* + 1) × (*W − m* + 1) with each pixel in the image (reference point to form the square window of length *m*); for example, if a 500 × 500 pixel image is processed, 6.1506 × 10^10^ comparison procedures are performed.

These extensive comparisons are a problem for many algorithms to quantify the irregularity of 2D data. This problem is so evident that the EntropyHub: Matlab platform (https://www.entropyhub.xyz/matlab/EHmatlab.html, accessed on 12 September 2021), which contains a repository of entropy algorithms, indicates in the documentation of some algorithms (for example, SampEn2D) a warning message related to the size of the images: “*…By default*, ‘*SampEn*2*D*’ only allows arrays with a maximum size of 128 × 128 to avoid RAM overhead… ** WARNING: unlocking the allowed array size can cause memory errors that could cause Matlab to crash **”.

The reduction of time in the 2D data processing, to quantify the irregularity, is a current problem that presents an interesting challenge for future proposals. Currently, the delay for processing in existing algorithms (including EspEn) is an impediment for real-time applications.

### 4.2. Shannon, SampEn2D, and EspEn Results (All Images)

**Figure 4:**
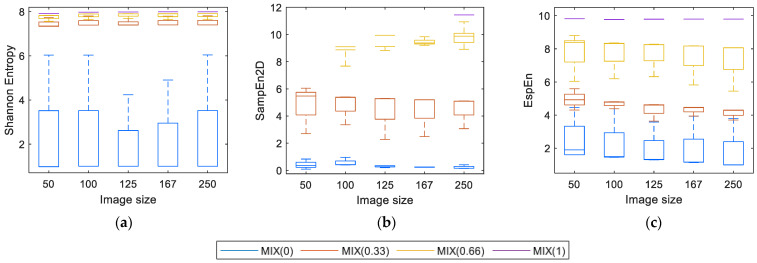
Values of entropy depending on the size: (**a**) Shannon, (**b**) SampEn2D, and (**c**) EspEn.

Figure 4 shows the entropy values of each algorithm applied to images with different degrees of contamination with the noise of uniform distribution and different sizes. Shannon Entropy (Figure 4a) can process regular images (MIX(0)) and irregular (MIX(1)). Nevertheless, the entropy values for MIX(0.33) and MIX(0.66) are very close to the maximum entropy value. Consequently, it is difficult to distinguish between images with different degrees of contamination. The resulting values do not vary in a relevant way regarding the variation in size and maintaining certain stability in the measurement.

Figure 4b shows the measurements of SampEn2D(*u*, *m*, *r*), where there is evidence of a problem already reported by da Silva et al. [24], related to the length of the square window (*m* = 2 or higher), for this case, the entropy values obtained were “Inf” for the MIX(1) images of size <250 × 250 pixels and for the images MIX(0.66) of size 50 × 50 pixels.

Figure 4c shows the EspEn results, where there is a distinction between images contaminated with different degrees of noise. The lowest entropy value was for regular images (MIX(0)), the highest value was for irregular images (MIX(1)) and intermediate values, clearly differentiated, for MIX(0.33) and MIX(0.66), being the values of MIX(0.66) > MIX(0.33). Entropy measurements were obtained with few variations according to the sizes evaluated.

### 4.3. EspEn Validation

This section presents the impact on the entropy measurements obtained with EspEn, varying the parameters *m*, *r*, and *ρ*, when the algorithm was applied to 100 × 100 pixel images contaminated with different degrees of white noise (MIX(*p*)).

#### 4.3.1. Dependence of EspEn on the Length of the Square Window (*m*)

**Figure 5:**
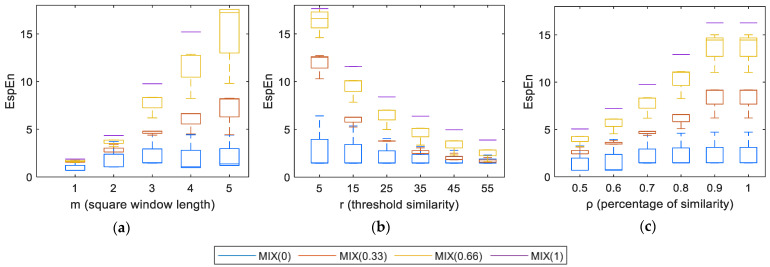
Dependence of EspEn on the parameters: (**a**) square window length (*m*), (**b**) threshold of similarity (*r*), and (**c**) percentage of similarity (ρ).

Figure 5a shows the behavior of the entropy measurements when *m* changes. A low value of *m* causes low entropy values for all MIX groups; this causes a difficult differentiation between the MIX groups. The increase in *m* allows a separation between the entropy values of the groups of MIX images, desirable for the classification between regular and irregular images. Table 1 shows that the most significant differences between MIX groups were obtained with *m* = 3 and *m* = 4.

#### 4.3.2. Dependence of EspEn with the Threshold of Similarity (*r*)

Figure 5b shows the effect of the variation of *r* on the entropy measurements, using EspEn. When *r* is low (a distance of five gray levels on the graph), there is a large separation between regular and irregular images, lower entropy values for MIX(0), and higher entropy values for MIX(1) and MIX(0.66). With the increase of *r*, the entropy values decrease for images with some degree of contamination by noise. When *r* is near or greater than the value of the standard deviation of the image, it is difficult to differentiate between MIX groups. At the other extreme, when *r* is very small (eg, *r* ≤ 1), differentiating between images contaminated with a high degree of noise (MIX (0.66) and MIX (1)) is difficult. Table 2 shows that the most significant differences between MIX groups were obtained with 15 < *r* < 35

#### 4.3.3. Dependence of EspEn with the Percentage of Acceptable Similarity (*ρ*)

Figure 5c shows the behavior of the entropy values regarding the change of ρ for images MIX. A low value of *ρ* causes low entropy values for all the MIX groups; this causes a difficult differentiation between the groups. The increase in *ρ* allows a separation between the entropy values of the groups of MIX images, as is desirable for the classification between regular and irregular images. Table 3 shows that, when *ρ* ≥ 0.7, the differentiation between the MIX groups improves.

### 4.4. Application of the EspEn Algorithm in the Images of Normalized Brodatz’s Texture Database

Although NBT images have been used in investigations of texture and irregularity analysis of two-dimensional data, there is no validated and accepted classification regarding the regularity or irregularity of each image in the database. Table 4 shows the entropy values obtained with the application of EspEn on NBT images, ordered from the lowest entropy value (regularity) to the highest entropy value (irregularity). This information can be a reference for new algorithm proposals to quantify the irregularity of an image.

**Figure 6:**
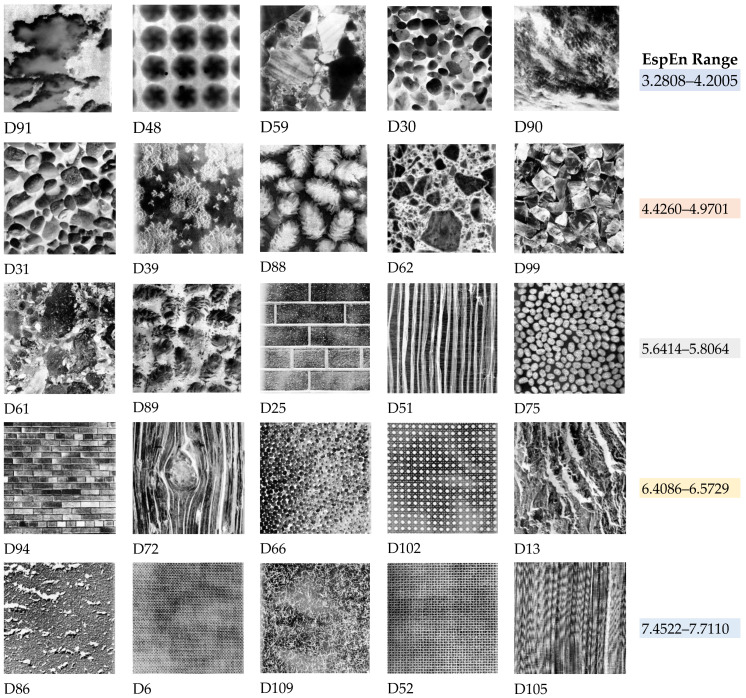

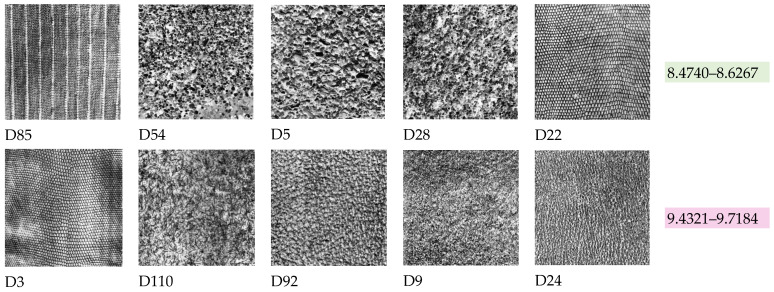
Database of Normalized Brodatz textures. The EspEn algorithm was applied to the images from https://multibandtexture.recherche.usherbrooke.ca/normalized_brodatz_more.html (accessed on 23 September 2021), and 5 images were taken as an example. The last column specifies the entropy value range obtained with EspEn for the images in the corresponding row. Each row can be interpreted as a degree of irregularity.

Figure 6 shows 35 NBT images as an example, distributed in five columns and seven rows; each row represents a range of entropy value, from greater regularity (first rows) to greater irregularity (last rows). For a better understanding, we use a code of color marking in Table 4 for the images and entropy values that we show in Figure 6. Column 6 of Figure 6 shows the Entropy range EspEn obtained from the images of the corresponding row.

### 4.5. Summary Characteristics of EspEn (u, m, r, ρ)

EspEn (*u*, *m*, *r*, ρ) is an innovative algorithm that allows users to quantify the irregularity present in an image. Parameter considerations to take into account include the image (*u*); image size is recommended to be low, due to computational cost, so that for large images (≥ 250 × 250 pixels) subsampling is performed. The value of *m* is recommended to be 2 ≤ *m* ≤ 4, typical value *m* = 3. The value of *r* is recommended to be 15 ≤ *r* ≤ 25, typical value *r* = 20 for std = ±80, avoid *r* ≥ std and *r* ≤ 1. The value of ρ is recommended to be 0.7 ≤ ρ ≤ 0.9, typical value ρ = 0.7. In case *m* ≥ 5 is used, decrease ρ-value if a weak similarity between windows is considered.

## 5. Conclusions

Entropy algorithms applied to images to estimate irregularity provide relevant information that can be used in texture analysis, classification, or segmentation processes. These algorithms have been useful in various fields of industry, agronomy, and biomedicine. We have proposed a new algorithm to quantify the irregularity of an image, called EspEn. The measurements provided by EspEn are consistent and robust for images contaminated with different degrees of noise. The following characteristics of EspEn stand out: (i) The entropy measurements show little variation with the change of the image size, overcoming the limitations that SampEn2D presents for small image sizes. (ii) The percentage of acceptable similarity (*ρ*) gives the researcher the possibility to decide how many pixels below *r* are accepted as a similarity between the pattern and the window. EspEn is more flexible than SampEn2D in the comparison between pattern and window. The rigidity of SampEn2D generates results that cannot be manipulated or interpreted. (iii) The similarity threshold takes into account the standard deviation but does not depend on it to control the limits allowed to perform the quantization of entropy without bias. (iv) EspEn is a simplified algorithm compared to SampEn2D, because it does not need to evaluate the algorithm in *m* + 1, and it is more robust than Shannon’s Entropy, because it takes into account spatial information from the image.

The most notable disadvantage of EspEn is the high computational cost for large images, which can be overcome by subsampling the image, because the entropy value does not differ greatly with the change in size.

## Figures and Tables

**Table 1:** Significance *p*-values between entropy values of MIX groups for different values of *m*.

	*p* Significance				
MIX Pairwise Comparisons	*m* = 1	*m* = 2	*m* = 3	*m* = 4	*m* = 5
MIX(0)–MIX(0.33)	0.260	0.392	0.108	0.085	0.023
MIX(0)–MIX(0.66)	0.024	0.007	0.001	0.001	0.000
MIX(0)–MIX(1)	0.000	0.000	0.000	0.000	0.023
MIX(0.33)–MIX(0.66)	0.260	0.069	0.077	0.085	--
MIX(0.33)–MIX(1)	0.001	0.000	0.001	0.001	--
MIX(0.66)–MIX(1)	0.021	0.054	0.087	0.085	--

**Table 2:** Significance *p*-values between entropy values of MIX groups for different values of *r*.

	*p* Significance					
MIX Pairwise Comparisons	*r* = 5	*r* = 15	*r* = 25	*r* = 35	*r* = 45	*r* = 55
MIX(0)–MIX(0.33)	0.083	0.134	0.392	0.392	0.392	0.392
MIX(0)–MIX(0.66)	0.000	0.001	0.003	0.003	0.007	0.007
MIX(0)–MIX(1)	0.000	0.000	0.000	0.000	0.000	0.000
MIX(0.33)–MIX(0.66)	0.051	0.069	0.032	0.032	0.069	0.069
MIX(0.33)–MIX(1)	0.001	0.000	0.000	0.000	0.000	0.000
MIX(0.66)–MIX(1)	0.193	0.087	0.087	0.087	0.054	0.054

**Table 3:** Significance *p*-values between entropy values of MIX groups for different values of *ρ*.

	*p* Significance					
MIX Pairwise Comparisons	*ρ* = 0.5	*ρ* = 0.6	*ρ* = 0.7	*ρ* = 0.8	*ρ* = 0.9	*ρ* = 1
MIX(0)–MIX(0.33)	0.454	0.392	0.108	0.087	0.085	0.085
MIX(0)–MIX(0.66)	0.005	0.003	0.001	0.001	0.001	0.001
MIX(0)–MIX(1)	0.000	0.000	0.000	0.000	0.000	0.000
MIX(0.33)–MIX(0.66)	0.042	0.032	0.077	0.087	0.085	0.085
MIX(0.33)–MIX(1)	0.000	0.000	0.001	0.001	0.001	0.001
MIX(0.66)–MIX(1)	0.069	0.087	0.087	0.087	0.085	0.085

**Table 4:** Entropy values EspEn obtained from the application of the algorithm EspEn in the images of Normalized Brodatz’s texture database.

Image	EspEn	Image	EspEn	Image	EspEn	Image	EspEn	Image	EspEn	Image	EspEn	Image	EspEn
D91	3.2809	D61	5.6415	D53	6.3611	D20	6.7323	D6	7.6567	D100	8.3514	D87	9.0954
D48	3.3013	D89	5.6994	D1	6.3648	D71	6.7439	D109	7.6722	D19	8.3903	D80	9.1871
D59	3.4067	D25	5.7179	D94	6.4086	D70	6.9054	D52	7.7059	D78	8.4588	D82	9.2503
D30	3.7778	D51	5.7569	D72	6.4134	D63	6.9277	D105	7.7110	D104	8.4685	D15	9.2758
D90	4.2005	D75	5.8065	D66	6.5300	D73	6.9387	D97	7.7605	D85	8.4740	D10	9.2975
D49	4.3454	D46	5.8070	D102	6.5414	D96	6.9952	D17	7.8667	D54	8.4931	D16	9.3094
D31	4.4261	D23	5.8098	D13	6.5729	D74	7.0016	D14	7.8877	D5	8.5038	D84	9.3123
D39	4.4927	D34	5.9653	D95	6.5874	D65	7.0283	D55	7.9221	D28	8.5315	D3	9.4322
D88	4.6406	D47	5.9830	D68	6.5995	D45	7.0507	D12	7.9348	D22	8.6267	D110	9.5855
D62	4.9174	D43	5.9947	D64	6.6246	D37	7.1197	D79	7.9453	D81	8.6805	D92	9.6326
D99	4.9702	D56	6.0135	D26	6.6279	D18	7.2808	D103	7.9758	D83	8.6998	D9	9.7136
D8	4.9785	D7	6.1287	D67	6.6363	D40	7.2836	D108	8.0863	D36	8.7392	D24	9.7185
D21	5.0191	D2	6.2263	D98	6.6713	D106	7.3473	D35	8.1938	D41	8.8204	D57	9.7451
D58	5.3044	D50	6.2309	D42	6.6808	D107	7.4336	D112	8.2544	D93	8.8927	D29	9.8173
D38	5.3089	D69	6.2565	D60	6.7170	D76	7.4341	D77	8.3207	D111	9.0168	D4	9.8291
D44	5.5531	D27	6.2585	D101	6.7309	D86	7.4523	D11	8.3302	D33	9.0535	D32	9.8896

## Data Availability

Not applicable.
